# Supplementary material for: Barriers to Optimal Child Sleep among Families with Low Income: A Mixed-Methods Study to Inform Intervention Development
Source: Int J Environ Res Public Health. 2023 Jan 3;20(1):862. doi: 10.3390/ijerph20010862 (PMC9820071; doi:10.3390/ijerph20010862)
Supplement: Supplementary file 1 [file ijerph-20-00862-s001.zip › ijerph-2064532-supplementary.docx]

**Supplementary File S1 (S1)**

**Interview script**

Hi {participant's name}. I am {interviewer's name}, and I'm excited to be interviewing you today. Thank you for talking with me about your child’s sleep. You input will be so valuable to us. The purpose of this interview is to learn more about how we can support families with young children in creating healthy sleep habits for their child and their whole family. This interview will help us to develop a program where we will teach and support parents in creating healthy sleep habits. For these interview questions, there are no right or wrong answers. We hope to make you feel comfortable sharing your personal experiences and perspective. Before we get started, I want to remind you that we will be audio-recording our conversation. This is only so that we can analyze the data later. Do you have any questions before we get started?

*[If yes: address participant questions / concerns.]*

*[If no: Okay, great, let’s go ahead and get started.]*

**1. Perceptions of bedtime, night wakings, morning routines, and naps**

Let’s start by thinking about your child’s bedtime.

Can you describe the activities typically done before bed, such as what they are, and how long they last?

How consistent are these same activities done before bed each night?

[if NOT consistent] What are some reasons these activities change night-to-night?

[if consistent] What are some factors that help you keep this consistency?

Do you have rules at bedtime around electronics or what time your child goes to bed?

How consistently does your child typically go to bed at the same time each night?

Prompt: For example, does bedtime change on days when your child does or does not attend childcare?

[if applicable] What are some reasons why bedtime differs on some nights?

Now let’s think about your child’s nighttime sleep.

Can you describe typical night wakings, such as how often the occur and how long they last?

Are there factors that seem to influence your child’s night wakings?

Prompt: For example, do you notice if there are more night wakings when certain things happen earlier that night?

What kinds of parenting strategies do you use to help your child fall back asleep?

Now let’s think about when your child typically wakes up in the morning.

Can you walk me through what typically occurs when your child wakes up?

How consistent is this morning routine day-to-day?

Prompt: For example, does this differ on days when your child does or does not attend childcare?

[if NOT consistent] What are some reasons these routines change day-to-day?

[if consistent] What are some factors that help you keep this consistency?

Now let’s think about your child’s typical daytime sleep.

Can you describe your child’s nap schedule, such as if they take naps, and the frequency/length of naps?

How consistent is your child’s typical nap schedule day-to-day?

Prompt: For example, does your child have different nap routines at home versus at daycare?

[if NOT consistent] What are some reasons why their nap schedule differs on some days?

[if consistent] What are some factors that help you keep this consistency?

[if applicable] What is your child’s nap environment like at childcare?

[if applicable] Do your child’s naps seem to impact their nighttime sleep? For example, on days they take naps, is bedtime or nighttime sleep different than days they do NOT take naps?

What are your overall feelings about the amount of sleep that your child gets?

Now let’s transition to talking about your home environment.

**2. Bedroom, home, and neighborhood environment**

Let’s think about the bedroom that your child typically sleeps in at night.

How would you describe this bedroom environment – such as the light, noise, or other family members that sleep in the same room (or the same bed) as your child?

How consistently does your child sleep typically sleep in this same environment?

If NOT consistently: Can you tell me about the other sleep environments? How often does your child change sleeping environments?

Think about the broader household and neighborhood environment where your child sleeps at night.

How would you describe your household environment – such as the noise, light, and the level of activity from other family members after your child goes to bed?

How would you describe your neighborhood environment – such as any street noise, lights, and crime safety?

Our next category focuses on challenges that you or other parents may face with sleep behaviors in your child.

**3. Sleep challenges**

Many parents struggle with their child’s sleep. Can you tell me about some struggles you experience related to your child’s sleep?

Does your work schedule, or the work schedule of other adults in your home, impact your child’s sleep schedule? How so?

What are some of the typical emotions you and your child experience around bedtime?

Prompt: For example, some parents feel happy like they are bonding with their child or frustrated and rushed getting their child to bed. What emotions do you experience?

To what extent do you think your child’s sleep impacts the sleep of other family members?

If you were to no longer experience challenges with your child’s sleep, how do you think that would impact your family’s emotional well-being?

Next, we want to learn about some effective strategies that you or other parents you know use to manage these sleep problems.

**4. Effective strategies**

What strategies do you find useful and effective when navigating challenges around children’s sleep?

What do you think would motivate parents to use new strategies to improve their child’s sleep?

What barriers may get in the way of parents using new strategies?

Prompt: For example, for some families, late work schedules, or feeling stressed and tired at night impacts their use of these strategies.

Now let’s think about the type of child sleep guidance you have received in the past.

**6. Receipt of current sleep guidance**

Where do you typically receive child sleep advice from, if at all?

[if applicable] What advice have you received about your child’s sleep?

Our last category will ask about your interest, or the interest of other parents you know, for a program to support healthy sleep habits. This is to help us inform the development of a sleep program to support parents.

**7. Intervention preferences**

How interested would you (or other parents you know) be in participating in a program that taught parents strategies to improve their children’s sleep?

If not interested: What are some reasons why you would not be interested?

How would you feel about this program being delivered individually, one-on-one or in a group setting?

Why is this format ideal for you?

How do you feel about where the program would occur (for example: remotely online, at a community center, in your home)?

Why is this setting ideal?

Is there anything else that you would like to share with us today about your child’s sleep?

**Supplementary File S2 (S2):**

| **Supplementary Table**. Median and interquartile range values for Child Sleep Wake Scale (CSWS) variables. Data from N=15 parents with a child 2-4 years of age. | | |
| --- | --- | --- |
|  | **Median** | **Interquartile range** |
| Duration of resisting bedtime (# min) | 45.0 | 45.0 |
| Duration to fall asleep at night (# min) | 60.0 | 78.0 |
| Night wakings (# times/night) | 2.0 | 2.0 |
| Duration to fall back asleep after waking (# min) | 10.0 | 15.0 |
| Duration to become alert in the mornning (# min) | 15.0 | 40.0 |
| CSWS subscales |  |  |
| Going to bed | 2.6 | 1.6 |
| Falling asleep | 3.0 | 2.0 |
| Maintaining sleep | 4.2 | 1.8 |
| Reinitiating sleep | 4.4 | 2.0 |
| Returning to wakefulness | 3.4 | 1.2 |
| CSWS total sleep quality score | 3.3 | 1.0 |
